# Feasibility study of peer-led and school-based social network Intervention (STASH) to promote adolescent sexual health

**DOI:** 10.1186/s40814-021-00835-x

**Published:** 2021-06-14

**Authors:** Kirstin R. Mitchell, Carrie Purcell, Sharon A. Simpson, Chiara Broccatelli, Julia V. Bailey, Sarah J. E. Barry, Lawrie Elliott, Ross Forsyth, Rachael Hunter, Mark McCann, Lisa McDaid, Kirsty Wetherall, Laurence Moore

**Affiliations:** 1 MRC/CSO Social and Public Health Sciences Unit, University of Glasgow, 99 Berkeley St, Glasgow, G3 7HR UK; 2 Institute for Social Science Research, The University of Queensland, Brisbane, Australia; 3 eHealth Unit, Research Department of Primary Care and Population Health, University College London, London, UK; 4 Department of Mathematics and Statistics, University of Strathclyde, Glasgow, UK; 5 Department of Nursing and Community Health, School of Health and Life Sciences, Glasgow Caledonian University, Glasgow, UK; 6 Robertson Centre for Biostatistics, Institute of Health and Wellbeing, University of Glasgow, Glasgow, UK

**Keywords:** Diffusion of innovation, Social network intervention, Sex education, School, Feasibility trial, Sexual health, Social media, Programme theory, Acceptability, Process evaluation, Young people, Adolescents, Non-randomised, Peer education, Peer support

## Abstract

**Background:**

Effective sex education is the key to good sexual health. Peer-led approaches can augment teacher-delivered sex education, but many fail to capitalise on mechanisms of social influence. We assessed the feasibility of a novel intervention (STASH) in which students (aged 14–16) nominated as influential by their peers were recruited and trained as Peer Supporters (PS). Over a 5–10-week period, they spread positive sexual health messages to friends in their year group, both in-person and via social media, and were supported to do so via weekly trainer-facilitated meetings. The aims of the study were to assess the feasibility of STASH (acceptability, fidelity and reach), to test and refine the programme theory and to establish whether the study met pre-set progression criteria for continuation to larger-scale evaluation.

**Methods:**

The overall design was a non-randomised feasibility study of the STASH intervention in 6 schools in Scotland. Baseline (*n*=680) and follow-up questionnaires (approx. 6 months later; *n*=603) were administered to the intervention year group. The control group (students in year above) completed the follow-up questionnaire only (*n*=696), 1 year before the intervention group. The PS (*n*=88) completed a brief web survey about their experience of the role; researchers interviewed participants in key roles (PS (*n*=20); PS friends (*n*=22); teachers (*n*=8); trainers (*n*=3)) and observed 20 intervention activities. Activity evaluation forms and project monitoring data also contributed information. We performed descriptive quantitative analysis and thematic qualitative analysis.

**Results:**

The PS role was acceptable; on average across schools >50% of students nominated as influential by their friends, signed up and were trained (*n*=104). This equated to 13% of the year group. Trained PS rarely dropped out (97% completion rate) and 85% said they liked the role. Fidelity was good (all bar one trainer-led activity carried out; PS were active). The intervention had good reach; PS were reasonably well connected and perceived as ‘a good mix’ and 58% of students reported exposure to STASH. Hypothesised pre-conditions, contextual influences and mechanisms of change for the intervention were largely confirmed. All bar one of the progression criteria was met.

**Conclusion:**

The weight of evidence supports continuation to full-scale evaluation.

**Trial registration:**

Current controlled trials ISRCTN97369178

**Supplementary Information:**

The online version contains supplementary material available at 10.1186/s40814-021-00835-x.

## Key messages regarding feasibility

1. **What uncertainties existed regarding the feasibility?**

- Would sufficient numbers of students nominated as ‘influential’ by their peers, be willing to take on and fulfil the peer supporter role? Would Peer Supporters have sufficient reach across year-group friendship networks? Would schools be willing and able to support the intervention?

2) **What are the key feasibility findings?**

- STASH was acceptable to Peer Supporters and students; activities were implemented with good fidelity and reached the majority of students; all bar one of the progression criteria were met with the weight of evidence supportive of continuation to a large-scale evaluation.

3) **What are the implications of the feasibility findings for the design of the main study?**

- Include activities to increase awareness of STASH activities across the year group, enable sharing from the STASH website to a wider range of social media platforms and consider delivering STASH to an older year group.

## Background

Reviews of intervention studies suggest that comprehensive sex education can be effective in delaying initiation of sex, reducing risky behaviour and increasing condom or contraceptive use [1, 2]. Survey research with adults also finds that citing school as a main source of learning about sex is associated with delaying first sex and avoiding unplanned pregnancy [3, 4]. However, globally, few children and young people receive adequate preparation for future sex lives in which they can make informed, free, positive and responsible choices [5]. In the UK, for instance, provision of good quality sex and relationship education (SRE) has been inconsistent [6], and over two thirds of young people in Britain report inadequate knowledge when they wished to have sex [7]. The social landscape for which young people must prepare is constantly changing, requiring effort to maintain the currency and relevance of sex education. A recent review of qualitative evidence highlighted that young people often find their school-based SRE out of touch, negative, gendered and heterosexist; and they dislike teacher-delivered SRE due to ‘blurred boundaries, lack of anonymity, embarrassment and poor training’ (pg 1) [8]. Given these limitations, there is a strong impetus for research to identify innovative ways to augment classroom learning.

Peer education offers opportunities to augment teacher-delivered SRE, though robust evidence of effectiveness in changing sexual behaviour is lacking [9, 10]. Peer education formally exploits naturally occurring communication channels across young people’s social networks, as well as the ‘insider knowledge’ that trained peer educators have of their own friendship cultures [11], and their credibility within them [12]. In theory, ongoing contact among peers of similar age and standing helps reinforce values, beliefs and social norms underpinning positive and healthy sexuality and sexual behaviour [12, 13]. In practice, interventions often fail to exploit the participatory, egalitarian and informal aspects of peer support [13]. Most peer-led approaches rely on self- or teacher-selection, resulting in educators who may be less credible and struggle to reach high-risk students [14]. Involving influential adolescent peers to spread and support healthy norms across their school-based social network is under-researched in sexual health, although there is growing evidence of the effectiveness of social network interventions more generally in sexual health [15].

Social media offer novel and innovative ways to transmit sexual health messages, rapidly and extensively [16–19]. As communication channels, social media and social networking sites are intuitively appealing given their popularity among young people. However, studies to date suggest caution, finding that young people are concerned with reputation management and may be reluctant to visibly engage with potentially stigmatising sexual health content online [20–24]. Few interventions successfully engage with the participatory aspects of social media or capitalise on young people’s expertise and knowledge to design approaches that resonate with young people’s everyday ‘practice’ of digital social interaction [23]. Possibly for these reasons, evidence of the effectiveness of social media interventions in improving sexual health outcomes among young people is mixed [16, 19, 25, 26]. The potential of social media combined with peer education in schools has not been explored [18].

Social network interventions commonly draw on diffusion of innovation theory [27, 28]. This posits that innovative ideas can be disseminated through a social network by influential members (‘early adopters’) of that network. There are four key elements: the innovation itself (in this case positive sexual attitudes and risk reduction), the channel of communication (in this case influential peers via conversation and social media), the differential response to the innovation (ranging from early enthusiasts to laggards) and the social system (in this case, school). The pace of adoption is said to be influenced by compatibility of the proposed innovation with existing values, the perceived relative advantage of adopting the new behaviour and the degree to which the new behaviours are straightforward to adopt, easy to try out and visible to others [28].

Diffusion of innovation theory underpinned the design of the STASH (Sexually Transmitted infections And Sexual Health) intervention, which recruited and trained influential students to disseminate positive sexual health messages, using social media as well as face-to-face conversation. We believe that the use of peer-led social media dissemination is a first for school-based sexual health interventions. We are aware of one other in-school social network intervention in sexual health using influential peers; the US-based STAND study [29], which used diffusion of innovation theory, through peer nomination to identify and train ‘opinion leaders’ in school. These opinion leaders were encouraged and supported to have one-on-one conversations with their peers about sexual risk reduction, with mixed impact on sexual attitudes and behaviour [30].

We present the results of a study to test the feasibility and acceptability of the STASH intervention and establish whether progression to a large-scale evaluation is warranted. The aims of the study necessitated a focus on evaluating process, and we followed the Medical Research Council guidance in exploring implementation (what was put in place and how was it implemented?), mechanisms of impact (how might the delivered activities produce change?) and context (how did the context shape implementation and outcomes?) [31]. We particularly focused on the latter, given that STASH is a complex intervention, with multiple interacting components and effects that vary according to the context in which they are delivered [32]. Our programme theory sought to capture this complexity, and we explicitly interrogated the theory as part of the feasibility trial [33].

## Methods

### The STASH intervention

The STASH intervention is based on diffusion of innovation theory [28] and is adapted from an effective peer-led anti-smoking intervention (ASSIST) in which ‘influential’ students (aged 12/13) were recruited via peer nomination and trained as Peer Supporters, to spread and sustain non-smoking norms through informal interactions [34]. STASH differed from ASSIST in three key ways: it focused on sexual health (rather than smoking), it targeted an older age group (14–16-year olds, not 12/13-year olds) and it utilised social media in addition to face-to-face conversations. STASH was co-produced with young people, experts in health and youth work, and intervention-delivery partners; it was piloted in one school [35]. The intervention is described in Table 1. The criteria for progression to full-scale evaluation are shown in table one. The full data against each criterion and analysis of key trial design parameters (including outcome measures) are presented in the main study report [36]. In this paper, we focus on 4 key areas of uncertainty regarding feasibility:

- Would students voted as ‘influential’ be willing to take on and fulfil the peer supporter role? [acceptability; progression criteria 1, 2b in Table 2]
- Would Peer Supporters be active and would they cope with the role? [fidelity; progression criterion 2a in Table 2]
- Would Peer Supporters reach students across the year group? [reach; progression criterion 2a in Table 2]
- Would schools be willing and able to support the intervention? [context; progression criteria 3b, 3c, 4 in Table 2]

**Table 1:** Description of STASH Intervention

The STASH intervention:	
**(1) Peer nomination.** All students in fourth year of secondary school (aged 14–16) asked to complete a peer nomination questionnaire, comprising 4 questions. A unique combination of questions is used in each school; two from the original three questions used in the ASSIST trial (who do you respect, who make good leaders, who do you look up to) and two drawn from four new questions designed for STASH (with whom do you feel comfortable talking about something personal, whose opinion do you trust, who is good at persuading others, who is confident at talking to people outside their friendship group). Top 25% of young people receiving most nominations, stratified by gender, invited to recruitment meeting.	
**(2) Peer Supporter (PS) recruitment meeting.** Trainers introduce STASH to the nominees, explain the PS role, and address questions. Aim is to recruit 15% of year group.	
**(3) Two-day PS training in school time, at external venue.** PS trained in knowledge, skills, confidence required for role. The training seeks to build motivation, enthusiasm, generate trust and rapport within PS group and between PS and trainers. PS sign a code of conduct agreement on completion of training, and agree plan to ‘announce’ the project to year group.	
**(4) Peer support work.** (a) PS establish ‘secret’ Facebook group (invite-only groups; highest privacy setting), comprising friends and STASH trainer. They post messages from the STASH website to this group and initiate face-to-face conversations centred on STASH messages. They alert friends to the STASH website and local support sources. PS are supported by a trainer and contact teacher. PS are encouraged to engage with STASH resources flexibly: for instance, in choosing which messages and links to share and editing messages into their own words if desired. (b) The trainers moderate group discussions, monitor Facebook posts, support the PS and facilitate follow-up meetings (weekly or fortnightly) with all PS.	
**(5) Acknowledgment of PS efforts.** Certificates, £10 voucher, ‘credit’ toward volunteering award.

**Table 2:** Summary of progression criteria to guide decision about whether to proceed to full-scale evaluation, sources of evidence for each criterion and whether targets were met

Green target^	Amber target	Red (targets not met)	Data source reference in text^^^^	Target met?
[1] Acceptability of role/feasibility: Was it feasible to recruit PS?*				
In at least 4 schools, 60% of nominated students recruited and complete the training.	50%, in at least 4 schools	Amber target achieved in fewer than 4 schools	Source 8; attendance at recruitment meeting	Red
**[2a] Reach/feasibility: Were PS able to carry out the role?**				
In ≥4 schools, 60% of PS complete training, send 3+ messages/have 3+ conversations and attend 2+ follow-up meetings	50%, in ≥4 schools	As above	Source 5; source 8	Green
**[2b] Acceptability: Was STASH acceptable to PS?**				
In ≥4 schools, 60% of PS report that they ‘liked’ the role	45%, in ≥4 schools.	As above	Source 5: ‘I liked being a peer supporter’ (5 point likert scale)	Green
**[3a] Acceptability: Was STASH acceptable to the wider target group?**				
In at least 4 schools, 60% of students who are exposed to STASH agree that the intervention was acceptable.	50%, in ≥4 schools.	As above	Source 2: ‘The way the STASH project was run/The information given in STASH was acceptable’ (2 items; 5-point likert scale)	Green
**[3b] Acceptability: Was STASH acceptable to participating schools?**				
No major acceptability issues raised^^^	1–2 major issues	Major acceptability issues	Source 6:Teachers	Green
**[3c] Acceptability: Was STASH acceptable to parents?**				
Less than 15% of PS report their parents/carers unhappy about them being a PS	<20%	Amber target not met	Source 5; Source 6	Green
**[4] Acceptability of evaluation/feasibility: Were the evaluation methods acceptable and feasible?**				
In at least 4 schools, student response rates of >70% at baseline and follow-up (FU)	Response of >60 in ≥4 schools	Amber target not met	Source 1,2,3 (Control, baseline and follow-up questionnaires); Source 6 (PS and non-PS interviews)	Green

*PS - Peer Supporters

^If green target met, this is taken as strong indication to proceed. Amber and red targets required discussion with the Trial Steering Committee and an identified mitigating strategy. In the case of a red, other indicators should be amber or green to proceed

^^Data sources are detailed in Table 3

^^^Major defined as an issue that threatened willingness of school to proceed with the intervention

Reproduced from Forsyth et al. [35]. This is an Open Access article distributed in accordance with the terms of the Creative Commons Attribution (CC BY 4.0) license, which permits others to distribute, remix, adapt and build upon this work, for commercial use, provided the original work is properly cited. See: http://creativecommons.org/licenses/by/4.0/. The table includes minor additions and formatting changes to the original table

### Study design

We present data from a non-randomised feasibility trial in six schools. The STASH intervention was delivered in all 6 schools during the first term of the academic year (August to December 2017). The baseline survey was administered to all fourth year students (including Peer Supporters) in August 2017 and a follow-up survey in March 2018. The control group comprised the previous year’s fourth year cohort in each school. They completed the follow-up survey only, at the same point in the academic year (March) but one calendar year previously (i.e. at the same age and school stage as the intervention group). Using the previous fourth year students as controls avoided the need to recruit additional schools for evaluation purposes only. Process data was collected during and after the intervention (see below). Process measures were collected across all 6 schools, and additional in-depth information was gathered from 4 ‘case-study’ schools. We report on progression criteria in relation to feasibility, acceptability, fidelity, reach and discuss whether theorised pre-conditions and mechanisms of change were observed. The protocol [35] and full report [36] describe the methods in detail.

### Recruitment of schools and students

Eligible participants for the intervention were students in their fourth year of high school (aged 14–16) who had previously received at least some teacher-led SRE (ascertained via school leadership teams), regardless of their sexual experience or individual level of risk. Private (fee-paying) schools or those in schools not currently providing comprehensive sex education were ineligible to join the STASH study.

All 17 state-funded schools in two education authorities were invited to participate; seven schools agreed and were recruited into the study (one took part in a pilot ahead of the main study; 6 took part in the main study). The 6 study schools varied in deprivation level (measured by proportion of students eligible for free school meals, ranging from 4.5 to 43.5%), size (measured by student headcount ranging from 279 to 1082) and location (large town, city outskirts and semi-rural). Head Teachers consented to school participation (via signed research agreement) on behalf of their students and teachers.

All students in the intervention year group were eligible for selection as Peer Supporters; however, only those nominated as ‘most influential’ by their peers were invited to recruitment sessions. Individual informed written consent was obtained for the Peer Supporter role.

Eligibility and recruitment of participants to each of the evaluation activities within the study varied by method and were determined by the purpose of the method.

### Study evaluation procedures

#### Control, baseline and follow-up questionnaire

These were web-based surveys administered to the entire fourth year group in school computer labs under exam conditions. The surveys were undertaken to assess the feasibility of in-school data collection methods (logistics, response rates), assess potential outcome/economic measures (e.g. reliability, missing data), identify potential modifiers (variables such as school engagement and peer risk behaviour that might affect exposure), capture year group social network (friendship) data and measure exposure to, and acceptability of, STASH activities.

#### Process evaluation questionnaires

All students and teachers attending the Peer Supporters training were asked to complete a brief evaluation form. At the final follow-up session, Peer Supporters were asked to complete a web-based questionnaire focusing on their experience of the role.

#### Semi-structured interviews

In 4 process evaluation case-study schools (purposively selected for variation in size, deprivation level and urban/rural location), Peer Supporters (*n*=20) and (separately) their friends (*n*=22) were interviewed in pairs or (single- and mixed-gender) groups. Peer Supporters were invited to participate in the interviews via the STASH contact teacher to give a mix of gender and engagement in the intervention. Friends of Peer Supporters were identified and invited to interviews via the Peer Supporters (with assistance from the contact teacher). Interviews were held in an empty classroom during a school period. They covered awareness of the STASH intervention, acceptability and engagement with messages and perceived impact. Interviews were also held with 8 senior staff and contact teachers covering the perceived value and impact of the intervention, and potential barriers and facilitators from the perspective of the school. All three STASH trainers were interviewed about their views on delivering STASH (what worked well and not so well).

#### Structured activity observations

were conducted on a sample of activities across the four case-study schools (4 recruitment sessions, 8 Peer Supporter training days and 8 follow-up sessions). A researcher observed and recorded notes on fidelity, acceptability, engagement, group dynamics and contextual factors.

Individual informed written consent was obtained for the web-based questionnaires and qualitative interviews (with opt-out parental consent).

Other data sources included a Peer Supporters activity monitoring log and project monitoring log (recording session attendance, correspondence with teachers, etc.). Table 3 links the methods to the source number.

**Table 3:** Summary of different methods (sample sizes and response rates) and their reference in the text

Reference in text	Method	Sample size/number of activities; response rates (questionnaires only)
Source 1	Baseline questionnaire	680/831 (80%)
Source 2	Follow-up questionnaire	603/744 (79%)
Source 3	Control questionnaire	696/864 (80%)
Source 4	Training evaluation	Completed by all students (*n*=104) and teachers (*n*=12) attending the training
Source 5	Peer Supporter Questionnaire	88 of 104 Peer Supporters (85%)
Source 6	Semi-structured interviews	Five group/paired interviews with PS (*n*=20) and 6 with PS friends (*n*=22); 7 interviews with 8 school staff (senior leaders and STASH contact teachers; Interviews with all STASH trainers (*n*=3)
Source 7	Activity observations	4 recruitment sessions, 8 training sessions and 8 follow-up sessions across 4 case-study schools.
Source 8	Monitoring log	n/a

### Key measures

#### Study progression criteria

The primary outcome of the trial was whether feasibility progression criteria were met. The seven criteria are summarised in Table 2.

#### Peer Supporter role completion

Trained Peer Supporters were recorded as completing the role if they (1) posted three or more STASH messages on Facebook OR had two or more face-to-face conversations about STASH and; (2) attended two or more follow-up sessions.

#### Intervention exposure

Students in the intervention year were categorised according to their exposure to the intervention as follows: (1) Peer Supporter, (2) exposed student (defined as reporting one or more of: being shown the STASH website by a Peer Supporters or accessing it themselves, joining a STASH Facebook group, talking with a Peer Supporters about a STASH topic) and (3) unexposed (not meeting criteria at (2)).

#### Social network measures

Baseline/follow-up questionnaires (source 1,2) asked students to name up to 6 friends with whom they spent time. Each named friend counted as a connection and these were used in 5 different social network measures to explore peer supporter position in the year group network: (1) Direct reach is the number of students directly connected to a Peer Supporter (% of year group); (2) In-degree is the number of incoming ties (i.e. frequency of being named as a friend); (3) Two-step reach centrality is the proportion of students connected to a Peer Supporters in two ‘steps’; (4) Eigenvector centrality indicates how ‘well-connected’ an individual is by considering how well-connected their friends are; (5) Target reach is the percentage of friendship clusters (using the Girvan-Newman algorithm) [37] containing a Peer Supporter.

### Analysis

#### Quantitative survey data

We present descriptive statistics. Consistent with the small sample size and exploratory analysis, percentages are rounded to the nearest whole number, and confidence intervals are not calculated.

#### Qualitative data

Transcribed data were entered into Nvivo 11 (QSR International, Warrington UK), to facilitate data management. We used a thematic-analytic approach was informed by the framework method [38, 39]. A coding framework (based on trial objectives) was applied by CP in discussion with KM. Following descriptive coding, data were interpreted to build themes and establish links between them.

#### Social network analysis (SNA)

In order to assess the potential reach of Peer Supporters (progression criteria 2a), we used network visualisation techniques and SNA measures to map and understand their position within year group networks, separately by school, using igraph [40] in R statistical software. We used the permutation-based *T* test procedure in UCINET6 [41] (inferential test for non-independent data) to examine differences in mean centralities between Peer Supporters and non-Peer Supporters.

### Ethical approval

The study was approved by the University of Glasgow MVLS Ethics committee (ref 200160002).

## Results and discussion

Findings and their interpretation are presented together for clarity, and for brevity, data sources are numbered. Table 3 shows the source number by method and presents sample sizes and response rates. All 6 recruited schools were retained in the trial. Questionnaires were completed by 680 intervention-year students (including the Peer Supporters) at baseline and 603 at follow-up (80% and 79% response rates, respectively). Of students completing baseline, 82% completed the follow-up. Since only three students opted out of the questionnaire, completion rates primarily reflected attendance at school on the day of survey administration. The green target (Table 2, criterion 4) was met for evaluation acceptability/feasibility.

### Testing the STASH programme theory

**Fig. 1:**
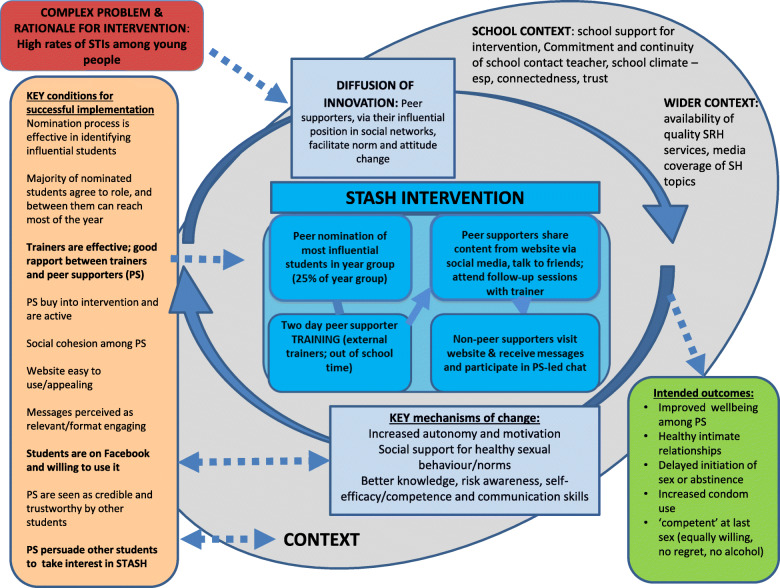
Simplified STASH programme theory, refined following feasibility study

The programme theory describes how intervention components interact, the mechanisms by which change occurs and the dynamic between context and intervention. A key objective for the feasibility study was to refine and test the programme theory and theoretical basis of the intervention. The programme theory was drafted at start of the study, elaborated during the development stage and interrogated during the feasibility trial [36]. The iteration of the programme theory at the main study stage is shown in the supplementary material. A simplified post-study Programme Theory (Fig. 1) shows the conditions, mechanisms of change and contextual factors confirmed as important by the process data. The macro (or grand) theory underpinning STASH was diffusion of innovation [28], and the feasibility questions below focus on dimensions of feasibility critical to successful diffusion. Key mechanisms of change were underpinned by behaviour change theories. For instance, in thinking about how best to motivate the Peer Supporters, we drew on self-determination theory which emphasises the importance of autonomy and intrinsic motivation [42]. The box in red (top left) describes the problem the intervention was designed to address. The bright blue box summarises the four key intervention components, encircled by the hypothesised mechanisms of change confirmed as important by the process data. The orange box to the left lists the key conditions that process data confirmed as critical for the mechanisms of change to work (particularly those in bold). These were met to a lesser or greater degree. The intervention took place within a broader school context (large grey circle), and again, aspects of the context identified as important are summarised. The intended outcomes are listed in the green box (bottom right). These were tested and clarified as part of the feasibility work. The feasibility questions below draw on and interrogate the programme theory.

### Acceptability: were influential students willing to take on and fulfil the Peer Supporter role?

An hypothesised pre-condition for STASH to be effective was that the majority of nominated students would agree to the role (i.e. that the role would seem acceptable to nominees). Our first green progression target (Table 2) was thus to recruit 60% of those nominated as ‘influential’ by their peers in at least 4 schools. In practice, this was challenging. We recruited >60% in one school, >50% in two schools and <50% in three (source 8). Although the criterion was not met, the groups of recruited Peer Supporters comprised 13% of their year groups overall (average across schools 13%, range 8 to 18%), just below the 15% level recommended by ASSIST (Campbell et al., 2008). The 60% uptake target was based on ASSIST (in which role-uptake was high (>90% of those nominated took up the role)) and may have been over-ambitious for STASH, given the topic, age and public-exam school year. The US-based STAND study also used peer nomination and had a similar uptake to STASH (50% of those nominated took up the role) [30].

Those who chose to attend the training were initially motivated by curiosity (27%), benefit to their curriculum vitae (21%) and having fun (19%; source 4), and the most common reason for completing the role was learning useful information (44% (*n*=68); source 5). These are primarily intrinsic motivations, suggesting that for the Peer Supporters at least, self-determination theory [42], was relevant to explaining our hypothesised mechanisms of change.

Once recruited, Peer Supporters rarely dropped out (97% completed the role). The majority of completers said they liked being a Peer Supporter (85% (73/86); 94% young women and 71% young men; source 5). Green progression targets 2a and 2b were thus both met (Table 2). Immediately after training, the most common fears held by Peer Supporters were not being listened to (30/99 post-coded text-box responses, source 4) or not taken seriously by their friends (18/99; source 4). At the end, 87% (76/87) said the training prepared them well, while 72% (63/87) felt confident in the role (source 5). Peer Supporter interviewees (source 6) thought the reasons others had not taken up the role were discomfort with the topic and disorganisation (e.g. not returning permission slips on time). Key reasons given for dwindling involvement were not wanting to miss classes (5 students) and frustration with Facebook/website technical problems (3 students; source 5).

We hypothesised that the rapport and trust between trainers and Peer Supporters would be a key condition of change. Our observation of activities (source 7), affirmed in interviews (source 6), suggested that a high level of rapport facilitated Peer Supporters engagement and role investment. Trainees valued the trainers being ‘good at having a laugh with us’ (Peer Supporters girls, source 6), and teachers observed that trainers were able to ‘get [the Peer Supporters] on board, to get them discussing’. (Contact teacher, source 6).

STASH was broadly acceptable to the wider year group. Of exposed students (*n*=268; source 2), 74% said the way STASH was run was acceptable, and 78% said the information provided was acceptable (source 2). Progression target 3a was thus met (Table 2).

### Fidelity: were Peer Supporters active and did they engage friends?

The fidelity of trainer-led activities was excellent with all trainings, and all except one follow-up activity, carried out as planned. The fidelity of Peer Supporters activities was also good. Of 104 trained Peer Supporters, 87 used Facebook to share STASH messages. The remaining 17 (mostly from a school in which Facebook use was low) used face-to-face conversation only. Facebook groups comprised an average of 12 members (including 7 who were not other Peer Supporters) and membership was stable. Peer Supporters were reasonably active, posting an average of 15 STASH-related messages each. They received an average of 9 relevant reactions (likes/comments/shares), indicating low-level overt online engagement by friends (source 8). Only 15 Peer Supporters directly messaged trainers. Most Peer Supporters (85%) reported at least 3 STASH topic conversations with friends, and 76% reported signposting friends to sources of help (source 5). Conversations included telling their friends about the activities they did in their training and signposting their friends to the resources on the STASH website.

Reactions to STASH posts on Facebook ranged from openness and interest to indifference (source 6). The STASH website was positively evaluated by the 175 students who said they visited (89% liked the way it looked; 79% found the information useful (source 2; note that website appeal was a key condition (Fig. 1)). Trainer monitoring of Peer Supporters Facebook groups was acceptable to peer supporters (92% of girls and 89% of boys glad or indifferent; source 5). The broader context—messages posted by an influential, trained student—appeared to legitimatise Facebook posts, but receptivity still varied. Preference for face-to-face conversation or social media communication varied, suggesting a mix of channels is appropriate (see also Hirvonen et al. [43]).

That non-Peer Supporters would regard Peer Supporters as trustworthy and credible was a hypothesised condition (Fig. 1). Formal training served to enhance Peer Supporters credibility: ‘But now that they’ve been taught about it […] you can listen to them a bit more, ‘cause […] they know what they’re talking about’ (female friend, source 6). Despite their credibility and enthusiasm, it was difficult for Peer Supporters to generate or sustain interest among their friends via brief messages and conversations. This may reflect variation in ‘readiness’ for messages about sexual health across the year group (around three quarters had not yet had oral or vaginal sex and may have decided the messages were not personally relevant) or lack of willingness to ‘admit’ interest.

Despite initial stakeholder fears of online bullying, no harms were reported. The most likely explanation was the presence of a STASH trainer in the online groups. Although close monitoring by trainers allayed fears and possibly prevented such incidents, it may also have stifled ‘natural’ engagement with STASH Facebook messages (see also Hirvonen et al. [43]).

There were small gender differences in Peer Supporters engagement. The gender split in role uptake (55% female) reflected the gender composition of the school years; and female Peer Supporters reported only slightly more activity than their male counterparts (source 5). However, female Peer Supporters were more likely to say they definitely or probably would keep sharing messages after STASH ended (43% versus 11%; source 5).

### Reach: did Peer Supporters reach students across the year group?

With respect to the peer supporter role, we hypothesised three conditions for STASH to be effective: (1) the nomination process would lead to the selection of students that were influential and representative of their year group and (2) the recruited Peer Supporters would reach most of the year between them (Fig. 1). These conditions were hypothesised based on learning from previous social network interventions using peer nomination [27] and on work by Borgatti and colleagues suggesting that Peer Supporter should optimally span the network [44].

We undertook social network analysis to determine how students were positioned in their network and whether they were better connected relative to students who were not nominated (or nominated but chose not to take up the role). Peer Supporters appeared better connected than other students (in-degree: 5.3 Peer Supporters were named as a friend (SD=2.4) versus 3.5 other students (SD=2.2); *p* value 0.0001 for two-tailed test). Similarly, 27% of students were connected to a Peer Supporter in two steps, compared with 20% for other students, (*p*=0.0001). However, there was no difference in eigenvector centrality scores (0.04 (SD=0.17) and 0.03 (SD=0.12) for Peer Supporters and other students respectively; *p* = 0.1677), suggesting no difference in the extent to which Peer Supporters and non-Peer Supporters were linked to ‘well-connected’ students in their network (source 2).

**Fig. 2:**
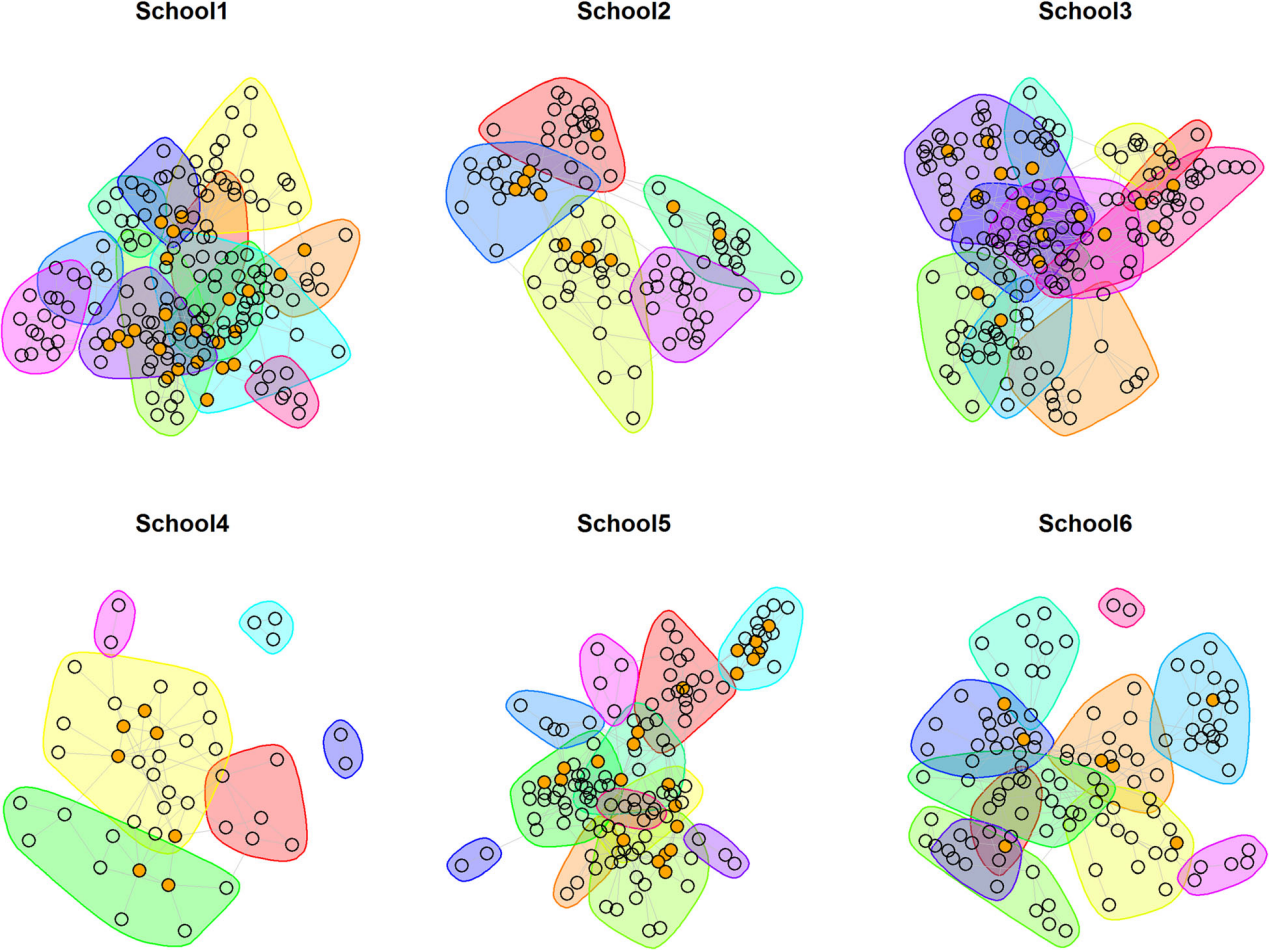
Distribution of PS within friendships clusters, by school. Key: nodes (circles) represent students; links among them indicate friendship ties. Friendship clusters (groups with many reciprocal ties) are highlighted in different colours; PS are orange dots*. Reproduced from Mitchell et al.* [36]. Copyright permission: Extracts (or indeed, the full report) may be included in professional journals provided that suitable acknowledgement is made and the reproduction is not associated with any form of advertising

We investigated the distribution of Peer Supporters across friendship clusters (groups with many reciprocal ties). The presence of clusters suggests segregation between groups and presents a potential barrier for message diffusion. Thus, Peer Supporters’ presence across many clusters is desirable. Figure 2 shows the proportion of clusters containing Peer Supporters varied between 33% and 80% (average 56%; source 2). This is consistent with findings from ASSIST [14], in which Peer Supporters were present in 50–60% of clusters. It suggests that, despite the older age group and more sensitive topic, Peer Supporters had reasonable potential to reach across their networks, with variation by school.

The direct reach measure indicates that on average, a third of students (34%, *N*= 302) were directly connected to a Peer Supporter. Schools 1 and 3 had the largest proportion of students directly connected to a Peer Supporters (52%), while the smallest proportion was in School 6 (7%).

**Fig. 3:**
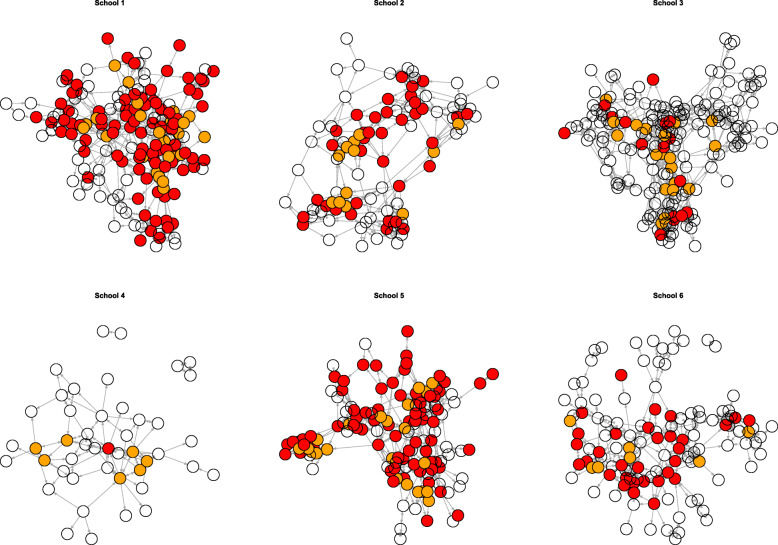
Peer Supporter distribution in Facebook groups by school. Key: PS are in orange, and friends who were at least in one Facebook group in red. *Reproduced from Mitchell et al.* [36]. Copyright permission: Extracts (or indeed, the full report) may be included in professional journals provided that suitable acknowledgement is made and the reproduction is not associated with any form of advertising

We also examined Peer Supporters friend connections in STASH Facebook groups (source 2; source 8). In Schools 1 and 5, 53% of students (excluding peer supporters) were members of a STASH Facebook group (respectively *n*=90 and *n*=71). In school 2, 37% (*N*= 37) of students were linked on Facebook, while in school 6 this was 25% (*N*=31). Few students were connected through Facebook in school 3 (9%, *N*=16) and school 4 (2%, *N*=1). This was because in school 3, Peer Supporters tended to use face-to-face interactions rather than Facebook and school 4 encountered challenges (contact teacher moved on; undergoing a significant transition), which seems reflected in minimal reach beyond the Peer Supporters (Fig. 3).

Peer Supporters were generally perceived as representative of their year group and well spread across the school: ‘there’s one [Peer Supporters] for every friend group’ (school 4, female friend); ‘a real mixed bag of pupils’ (teacher; source 6).

In terms of exposure to the intervention (Table 4; source 1,2), those recruited as Peer Supporters were similar to other students in terms of socio-economic status (Scottish Index of Multiple Deprivation (SIMD) quintile [45], receipt of free school meals (an indicator of family income), and home ownership). Peer Supporters reported the highest academic attainment (followed by exposed then unexposed students) but seemed no more likely to say they ‘tried hard’ at school. There seemed little difference by ethnicity, religiosity and sexual orientation, but young women were more likely to report exposure than young men (62% of exposed were female). There was some suggestion of a gradient by sexual experience at baseline, suggesting the intervention may have reached students for whom the messages were most relevant.

**Table 4:** Baseline characteristics reported by Peer Supporters, students (excluding peer supporters) who reported exposure to one or more intervention components and students who reported no exposure (includes only students for whom baseline and follow-up data available)

***N*** (%)		PS	Exposed	Unexposed
**Demographics**				
**Gender**	*N*_obs_ (*N*_miss_) Male Female Other	97 (0) 41 (42%) 55 (57%) 1 (1%)	240 (1) 87 (36%) 150 (63%) 3 (1%)	222 (2) 103 (46%) 112 (51%) 7 (3%)
**SIMD quintile**	*N*_obs_ (*N*_miss_) 1 - Most deprived 2 3 4 5 - Least deprived	72 (25) 9 (12%) 15 (21%) 9 (12%) 18 (25%) 21 (29%)	172 (68) 25 (14%) 38 (22%) 33 (19%) 29 (17%) 47 (27%)	144 (78) 17 (12%) 27 (18%) 23 (16%) 27 (19%) 50 (35%)
**Free school meal eligible**	*N*_obs_ (*N*_miss_) No Yes	97 (0) 82 (84%) 15 (16%)	240 (1) 219 (91%) 21 (9%)	221 (3) 197 (89%) 24 (11%)
**Residence type**	*N*_obs_ (*N*_miss_) House/flat owned by family Other	97 (0) 69 (71%) 28 (28%)	239 (2) 168 (70%) 71 (30%)	221 (3) 157 (71%) 64 (29%)
**Exam level studying for**^**1**^	*N*_obs_ (*N*_miss_) National 5 only National 4 only or 4 & 5	94 (0) 79 (84%) 15 (16%)	222 (0) 160 (72%) 62 (28%)	194 (0) 135 (70%) 69 (30%)
**Religiosity**	*N*_obs_ (*N*_miss_) Very/quite important Not important	95 (2) 12 (13%) 83 (87%)	237 (4) 32 (14%) 205 (86%)	220 (4) 35 (16%) 185 (84%)
**Ethnicity**	*N*_obs_ (*N*_miss_) White Scottish/British White but not Scottish/British Asian African/Caribbean/Black Other/Mixed	97 (0) 92 (95%) 2 (2.1%) 1 (1%) 2 (2%) 0 (0%)	239 (2) 220 (92%) 9 (4%) 5 (2%) 1 (0%) 4 (2%)	222 (2) 195 (88%) 10 (4%) 8 (4%) 3 (1%) 6 (3%)
**Sexual identity**	*N*_obs_ (*N*_miss_) Heterosexual/straight Gay or lesbian Bisexual Other	97 (0) 88 (91%) 3 (3%) 3 (3%) 3 (3%)	240 (1) 215 (90%) 6 (2%) 10 (4%) 9 (4%)	219 (5) 191 (87%) 6 (3%) 12 (6%) 10 (4%)
**Sexual experience**	*N*_obs_ (*N*_miss_) None Kissing/touching genitals Oral or vaginal sex	91 (6) 39 (43%) 32 (35%) 20 (22%)	242 (0) 115 (48%) 85 (35%) 42 (17%)	229 (0) 120 (52%) 62 (27%) 47 (21%)
**Connectedness and engagement with school**				
**I feel close to people in the school**	*N*_obs_ (*N*_miss_) Agree Disagree	97 (0) 80 (82%) 17 (18%)	241 (0) 187 (78%) 54 (22%)	220 (4) 173 (79%) 47 (21%)
**I try hard in school**	*N*_obs_ (*N*_miss_) Agree Disagree	97 (0) 86 (89%) 11 (11%)	240 (1) 224 (93%) 16 (7%)	220 (4) 201 (91%) 19 (9%)

^1^More academic students usually study 6 or more subjects at national 5 level; less academic students usually study a mix of national 4 and national 5 level subjects

*Reproduced from Mitchell et al.* [36]. Copyright permission: Extracts (or indeed, the full report) may be included in professional journals provided that suitable acknowledgement is made and the reproduction is not associated with any form of advertising

### Support: were schools willing and able to support the intervention?

Most school staff were positive about STASH, and no major acceptability issues arose (green progression target 3b met; Table 2). They valued the leadership opportunities and the ethos of openness about sexual matters (source 6); they were impressed by the rapport between trainers and Peer Supporters: the trainers had ‘a really good handle on the kids’, who ‘would have felt that they were respected and listened to.’ Another noted the excellent quality of delivery that enabled trainers to ‘get a lot of information into a short space of time’ (Teacher, source 6).

Teachers in position to compare STASH with school SRE (about half the sample) generally saw it as offering an update to out-of-date materials. The relatively relaxed STASH environment was also highlighted as facilitating ‘sensible questions […] about risk-taking behaviour’. One teacher reflected: ‘they certainly wouldn’t ask me those questions’. (Teacher, source 6).

Regarding broader impact, teachers noted the combined growth in ‘young people’s awareness about sex education’, alongside the fact that STASH had ‘developed confidence [and] given them an opportunity to develop leadership skills’ (Teacher, source 6). The benefit of improved confidence was particularly highlighted in School 5, with a teacher noting ‘how articulate, vocal’ one male Peer Supporter had become, and highlighting improvements in wider skills and confidence across the group.

At the same time, staff perceived extra workload for them (both for the intervention and evaluation), and some expressed concern that STASH activities meant students losing class time during an exam year (teacher, source 6). There was a fairly consistent view across teacher interviews that, whatever attempts might be made, fourth year is challenging to fit in additional activities, and that the ‘big commitment’ required took ‘a big chunk out of their [exam subject] lessons that they then have to catch up on’ (Teacher, source 6). Of eight staff members interviewed, only one (school 4) raised concerns; these related primarily to their capacity to participate, as they were undergoing a major transition.

The variability across schools in uptake and engagement was largely consistent with the level of school support for the intervention. For instance, the highest role uptake followed a recruitment session at which a head teacher was present, and the most active group of peer supporters were supported by a teacher who held a pastoral (non-teaching) role and could offer more time. In two schools, the project was passed to a new contact teacher due to ill health or changing responsibilities. The researchers had little control over how well these transitions were managed, but in both cases, they negatively influenced implementation.

Acceptability to parents/carers was assessed indirectly via Peer Supporters. Only one student reported that their parent/carer was unhappy about them being a Peer Supporter (source 5). Green progression target 3c was thus met. The evaluation did not identify any unintended harmful effects of the intervention.

### (Re)Interrogating the programme theory

Programme theories provide a framework for understanding implementation uncertainties and should be continually refined as feasibility work progresses. The evolution from the intervention implementation stage (Supplementary material one) to analysis of findings (Fig. 1) reflects learning from the process evaluation. Together, the data described above largely confirmed that the programme theory adequately captured the conditions of success for STASH and that these conditions were largely met, albeit with variation across schools. In terms of the hypothesised mechanisms of change, our data largely confirmed these with a few exceptions. We envisaged that social validation would be a key mechanism (supplementary material). In practice, we found that the Peer Supporters did not appear to be socially validated in their role (they were neither validated nor disapproved of), yet the role still appeared to enhance self-esteem and wellbeing. Thus, we removed this mechanism from the final programme theory (Fig. 1). Being nominated as an influential peer was a key mechanism in generating Peer Supporter confidence and self-esteem, confirming additional benefit of peer-nomination over other social network criteria (e.g. selecting individuals that optimally span the network) [27, 44]. Our finding that it was difficult for Peer Supporters to generate high levels of engagement among non-Peer Supporters prompted reflection on whether it was realistic to expect the Peer Supporters to generate intrinsic motivation to engage with the intervention among their friends and whether this was a key mechanism of change; this mechanism was also removed. It is possible that information and norm change could still diffuse through a network without overt individual online engagement by Peer Supporter friends, although this remains an uncertainty.

### Were study progression criteria met?

All study progression criteria were met with ease, except the first (60% uptake of Peer Supporters role in 4 or more schools). In discussion with the Trial Steering Committee, it was agreed that failure to meet this target did not negate continuation since the trial was still able to recruit 13% of the year group and recruited Peer Supporters were well positioned across their network. Nonetheless, mitigation strategies should be considered.

### Limitations of the study

As a non-randomised and small-scale feasibility study, STASH had several limitations. The sample of 6 schools was never intended to indicate effectiveness, but it was also too small to detect significant differences in process indicators such as differential exposure by gender or educational attainment. The constraints of conducting qualitative fieldwork within class periods meant that there was insufficient time to build a strong rapport between researchers and students and to cover all topics in depth. We detected a reticence to discuss sexual health topics with a researcher in a school setting, and it was sometimes difficult to tell whether lack of recall about the intervention stemmed from minimal engagement or reluctance to admit to engagement in front of friends. We were unable to access parents or to follow-up peer supporters who dropped out; their views were measured indirectly via teachers and completing peer supporters. Finally, the research team was involved both in the design, implementation and delivery of STASH and this non-independence needs to be taken into account in the interpretation of results.

### Future refinements

Our data suggest some small-scale refinements to intervention components and programme theory are advisable. For instance, awareness and engagement might be increased by an initial SRE session to the year group, in which trainers explain the project and introduce the Peer Supporters. Enabling sharing from the STASH website to a wider range of social media platforms would also be helpful, given limited Facebook use. Delivering STASH to an older year group, with a greater number of free periods might increase student engagement and acceptability to schools. STASH could reinforce prior classroom-based SRE.

## Conclusions

The STASH intervention offers an innovative approach to SRE which exploits mechanisms of social influence to spread norm and attitude change. It is novel in engaging peer-led use of social media in conjunction with face-to-face conversation. The intervention appears broadly feasible and acceptable in Scottish secondary schools, and there is evidence of broader benefits (Peer Supporter confidence and skills) beyond sexual health. The pre-conditions, key contextual influences and mechanisms of change hypothesised in the programme theory are largely confirmed by process data.

The conditions of success and mechanisms of change identified for STASH are generic, and the process data suggest that STASH should be broadly feasible and acceptable to any school that is supportive of comprehensive sex and relationships education. The approach has inbuilt flexibility for adaption to different school timetables and curricula and could be conducted in any senior school year (from age 14). Our feasibility trial suggests that years with less exam pressure are preferable.

The weight of evidence from this study supports continuation to full-scale evaluation. The approach is empowering to young people and supportive of their right to quality SRE, as well as to actively participate and shape their learning.

## Supplementary Information

**Additional file 1.** Supplementary material. Programme Theory at time of Intervention Implementation

## Data Availability

The full report of the STASH study is available [36]. Excerpts of raw qualitative data are available on reasonable request from the authors. The questionnaire data is available on request via the University of Glasgow Enlighten repository (https://www.gla.ac.uk/research/enlighten/).

## Abbreviations

PS: Peer supporter
SNA: Social network analysis
SRE: Sex and relationship education

## References

[1] Kirby DB. The impact of abstinence and comprehensive sex and STD/HIV education programs on adolescent sexual behavior. Sex Res Social Policy. 2008;5(3):18–27. 10.1525/srsp.2008.5.3.18.

[2] Kirby DB, Laris B, Rolleri LA. Sex and HIV education programs: their impact on sexual behaviors of young people throughout the world. J Adolesc Health. 2007;40(3):206–17. 10.1016/j.jadohealth.2006.11.143. 10.1016/j.jadohealth.2006.11.143 17321420

[3] Macdowall W, Jones KG, Tanton C, Clifton S, Copas AJ, Mercer CH, et al. Associations between source of information about sex and sexual health outcomes in Britain: findings from the third National Survey of Sexual Attitudes and Lifestyles (Natsal-3). BMJ open. 2015:5(3). 10.1136/bmjopen-2015-007837. 10.1136/bmjopen-2015-007837 PMC4360826 25743154

[4] Barrense-Dias Y, Akre C, Surís J-C, Berchtold A, Morselli D, Jacot-Descombes C, et al. Does the primary resource of sex education matter? A Swiss national study. J Sex Res. 2020;57(2):166–76. 10.1080/00224499.2019.1626331. 10.1080/00224499.2019.1626331 31215800

[5] UNESCO, UNAIDS, UNFPA, UNICEF, UN Women and WHO. International technical guidance on sexuality education: an evidence-informed approach: UNESCO Publishing; 2018. https://www.unfpa.org/publications/international-technical-guidance-sexuality-education. Accessed 5 May 2021.

[6] Ofsted. Not yet good enough: personal, social, health and economic education in schools. The Office for Standards in Education, Children’s Services and Skills, Manchester, England; 2013. www.ofsted.gov.uk/resources/130065.

[7] Tanton C, Jones KG, Macdowall W, Clifton S, Mitchell KR, Datta J, et al. Patterns and trends in sources of information about sex among young people in Britain: evidence from three National Surveys of Sexual Attitudes and Lifestyles. BMJ open. 2015;5:e007834. 10.1136/bmjopen-2015-007834. 10.1136/bmjopen-2015-007834 PMC4360842 25743153

[8] Pound P, Langford R, Campbell R. What do young people think about their school-based sex and relationship education? A qualitative synthesis of young people’s views and experiences. BMJ open. 2016;6:e011329. 10.1136/bmjopen-2016-011329. 10.1136/bmjopen-2016-011329 PMC5030567 27625058

[9] Sun WH, Miu HYH, Wong CKH, Tucker JD, Wong WCW. Assessing participation and effectiveness of the peer-led approach in youth sexual health education: systematic review and meta-analysis in more developed countries. J Sex Res. 2018;55(1):31–44. 10.1080/00224499.2016.1247779. 10.1080/00224499.2016.1247779 27898248

[10] Kim CR, Free C. Recent evaluations of the peer-led approach in adolescent sexual health education: a systematic review. Perspect Sex Reprod Health. 2008;40(3):144–51. 10.1363/4014408. 10.1363/4014408 18803796

[11] Milburn K. A critical review of peer education with young people with special reference to sexual health. Health Educ Res. 1995;10(4):407–20. 10.1093/her/10.4.407. 10.1093/her/10.4.407 10159674

[12] Tolli MV. Effectiveness of peer education interventions for HIV prevention, adolescent pregnancy prevention and sexual health promotion for young people: a systematic review of European studies. Health Educ Res. 2012;27(5):904–13. 10.1093/her/cys055. 10.1093/her/cys055 22641791

[13] Harden A, Oakley A, Oliver S. Peer-delivered health promotion for young people: a systematic review of different study designs. Health Educ J. 2001;60(4):339–53. 10.1177/001789690106000406.

[14] Starkey F, Audrey S, Holliday J, Moore L, Campbell R. Identifying influential young people to undertake effective peer-led health promotion: the example of A Stop Smoking In Schools Trial (ASSIST). Health Educ Res. 2009;24(6):977–88. 10.1093/her/cyp045. 10.1093/her/cyp045 19684123

[15] Hunter RF, de la Haye K, Murray JM, Badham J, Valente TW, Clarke M, et al. Social network interventions for health behaviours and outcomes: a systematic review and meta-analysis. PLoS Med. 2019;16(9):e1002890. 10.1371/journal.pmed.1002890. 10.1371/journal.pmed.1002890 PMC6719831 31479454

[16] Gabarron E, Wynn R. Use of social media for sexual health promotion: a scoping review. Global Health Action. 2016;9(1):32193. 10.3402/gha.v9.32193. 10.3402/gha.v9.32193 PMC5030258 27649758

[17] Swanton R, Allom V, Mullan B. A meta-analysis of the effect of new-media interventions on sexual-health behaviours. Sex Transm Infect. 2015;91(1):14–20. 10.1136/sextrans-2014-051743. 10.1136/sextrans-2014-051743 25433051

[18] Wadham E, Green C, Debattista J, Somerset S, Sav A. New digital media interventions for sexual health promotion among young people: a systematic review. Sex Health. 2019;16(2):101–23. 10.1071/SH18127. 10.1071/SH18127 30819326

[19] Jones K, Eathington P, Baldwin K, Sipsma H. The impact of health education transmitted via social media or text messaging on adolescent and young adult risky sexual behavior: a systematic review of the literature. Sexually Transmitted Dis. 2014;41(7):413–9. 10.1097/OLQ.0000000000000146. 10.1097/OLQ.0000000000000146 24922099

[20] Veinot TC, Campbell TR, Kruger D, Grodzinski A, Franzen S. Drama and danger: the opportunities and challenges of promoting youth sexual health through online social networks. AMIA Annu Symp Proc. 2011;2011:1436-45. PMC3243290 22195207

[21] Byron P, Albury K, Evers C. “It would be weird to have that on Facebook”: young people’s use of social media and the risk of sharing sexual health information. Reprod Health Matters. 2013;21(41):35–44. 10.1016/S0968-8080(13)41686-5. 10.1016/S0968-8080(13)41686-5 23684185

[22] Ralph LJ, Berglas NF, Schwartz SL, Brindis CD. Finding teens in TheirSpace: using social networking sites to connect youth to sexual health services. Sexual Res Soc Policy. 2011;8(1):38–49. 10.1007/s13178-011-0043-4.

[23] Byron P. Troubling expertise: social media and young people’s sexual health. Commun Res Pract. 2015;1(4):322–34. 10.1080/22041451.2015.1110085.

[24] Nadarzynski T, Burton J, Henderson K, Zimmerman D, Hill O, Graham C. Targeted advertisement of chlamydia screening on social media: a mixed-methods analysis. Digital health. 2019;5:2055207619827193. 10.1177/2055207619827193 PMC6360644 30746155

[25] Swanton R, Allom V, Mullan B. A meta-analysis of the effect of new-media interventions on sexual-health behaviours. Sexual Transmitted Infect. 2015;91(1):14–20. 10.1136/sextrans-2014-051743. 10.1136/sextrans-2014-051743 25433051

[26] Wadham E, Green C, Debattista J, Somerset S, Sav A. New digital media interventions for sexual health promotion among young people: a systematic review. Sexual Health. 2019;16(2):101–23. 10.1071/SH18127. 10.1071/SH18127 30819326

[27] Valente TW. Network interventions. Science. 2012;337(6090):49–53. 10.1126/science.1217330. 10.1126/science.1217330 22767921

[28] Rogers EM. Diffusion of preventive innovations. Addict Behav. 2002;27(6):989–93. 10.1016/S0306-4603(02)00300-3. 10.1016/s0306-4603(02)00300-3 12369480

[29] Smith MU, DiClemente RJ. STAND: a peer educator training curriculum for sexual risk reduction in the rural south. Prev Med. 2000;30(6):441–9. 10.1006/pmed.2000.0666. 10.1006/pmed.2000.0666 10901486

[30] Smith MU, Dane FC, Archer ME, Devereaux RS, Katner HP. Students together against negative decisions (STAND): evaluation of a school-based sexual risk reduction intervention in the rural south. AIDS Educ Prev. 2000;12(1):49–70. 10749386

[31] Moore GF, Audrey S, Barker M, Bond L, Bonell C, Hardeman W, et al. Process evaluation of complex interventions: Medical Research Council guidance. British Med J. 2015;350:1–6. 10.1136/bmj.h1258. 10.1136/bmj.h1258 PMC4366184 25791983

[32] Hawe P. Lessons from complex interventions to improve health. Ann Rev Public Health. 2015;36(1):307–23. 10.1146/annurev-publhealth-031912-114421. 10.1146/annurev-publhealth-031912-114421 25581153

[33] Rogers PJ. Using programme theory to evaluate complicated and complex aspects of interventions. Evaluation. 2008;14(1):29–48. 10.1177/1356389007084674.

[34] Campbell R, Starkey F, Holliday J, Audrey S, Bloor M, Parry-Langdon N, et al. An informal school-based peer-led intervention for smoking prevention in adolescence (ASSIST): a cluster randomised trial. Lancet. 2008;371(9624):1595–602. 10.1016/S0140-6736(08)60692-3. 10.1016/S0140-6736(08)60692-3 PMC2387195 18468543

[35] Forsyth R, Purcell C, Barry S, Simpson S, Hunter R, McDaid L, et al. Peer-led intervention to prevent and reduce STI transmission and improve sexual health in secondary schools (STASH): protocol for a feasibility study. Pilot Feasibility Stud. 2018;4(1):180. 10.1186/s40814-018-0354-9. 10.1186/s40814-018-0354-9 PMC6264037 30519482

[36] Mitchell KR, Purcell C, Forsyth R, Barry S, Hunter R, Simpson SA, et al. A peer-led intervention to promote sexual health in secondary schools: the STASH feasibility study. Public Health Res. 2020;8(15):ISSN 2050-4381. 10.3310/phr08150. 33252893

[37] Snijders TA, Van de Bunt GG, Steglich CE. Introduction to stochastic actor-based models for network dynamics. Soc Networks. 2010;32(1):44–60. 10.1016/j.socnet.2009.02.004.

[38] Gale NK, Heath G, Cameron E, Rashid S, Redwood S. Using the framework method for the analysis of qualitative data in multi-disciplinary health research. BMC Med Res Methodol. 2013;13(1):1–8. 10.1186/1471-2288-13-117. 10.1186/1471-2288-13-117 PMC3848812 24047204

[39] Ritchie J, Lewis J, Nicholls CM, Ormston R. Qualitative research practice: a guide for social science students and researchers. London: Sage; 2013.

[40] Csardi G, Nepusz T. The igraph software package for complex network research. InterJ Complex Sys. 2006;1695(5):1–9. https://igraph.org/. Accessed 5 May 2021.

[41] Borgatti SP, Everett MG, Freeman L. Ucinet for Windows: Software for Social Network Analysis. Harvard: Analytic Technologies; 2002. https://sites.google.com/site/ucinetsoftware/home. Accessed 5 May 2021.

[42] Ryan RM, Deci EL. Self-determination theory and the facilitation of intrinsic motivation, social development, and well-being. Am Psychol. 2000;55(1):68–78. 10.1037/0003-066X.55.1.68. 10.1037//0003-066x.55.1.68 11392867

[43] Hirvonen M, Purcell C, Elliott L, Bailey JV, Simpson SA, McDaid L, et al. Peer-to-Peer Sharing of Social Media Messages on Sexual Health in a School-Based Intervention: Opportunities and Challenges Identified in the STASH Feasibility Trial. J Med Internet Res. 2021;23(2):e20898. 10.2196/20898. 10.2196/20898 PMC7925155 33591287

[44] Borgatti SP. Identifying sets of key players in a social network. Computat Mathematical Org Theory. 2006;12(1):21–34. 10.1007/s10588-006-7084-x.

[45] Scottish Index of Multiple Deprivation 2020. https://www.gov.scot/collections/scottish-index-of-multiple-deprivation-2020. Accessed 5 May 2021.

