# Supplementary material for: Feasibility study of peer-led and school-based social network Intervention (STASH) to promote adolescent sexual health
Source: Pilot Feasibility Stud. 2021 Jun 14;7:125. doi: 10.1186/s40814-021-00835-x (PMC8201683; doi:10.1186/s40814-021-00835-x)
Supplement: Supplementary file 1 — Additional file 1. Supplementary material. Programme Theory at time of Intervention Implementation [file 40814_2021_835_MOESM1_ESM.docx]

**
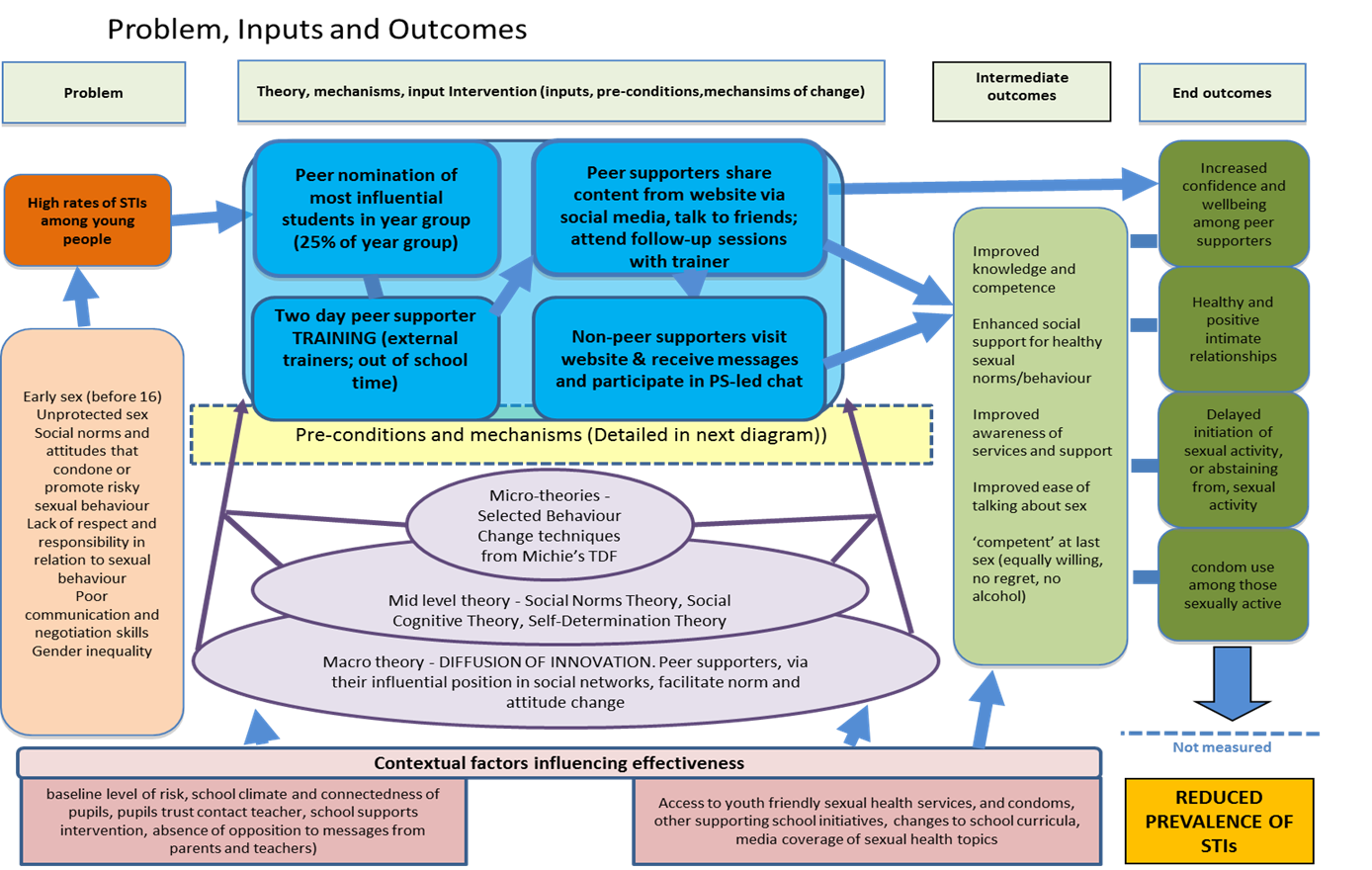
**

**
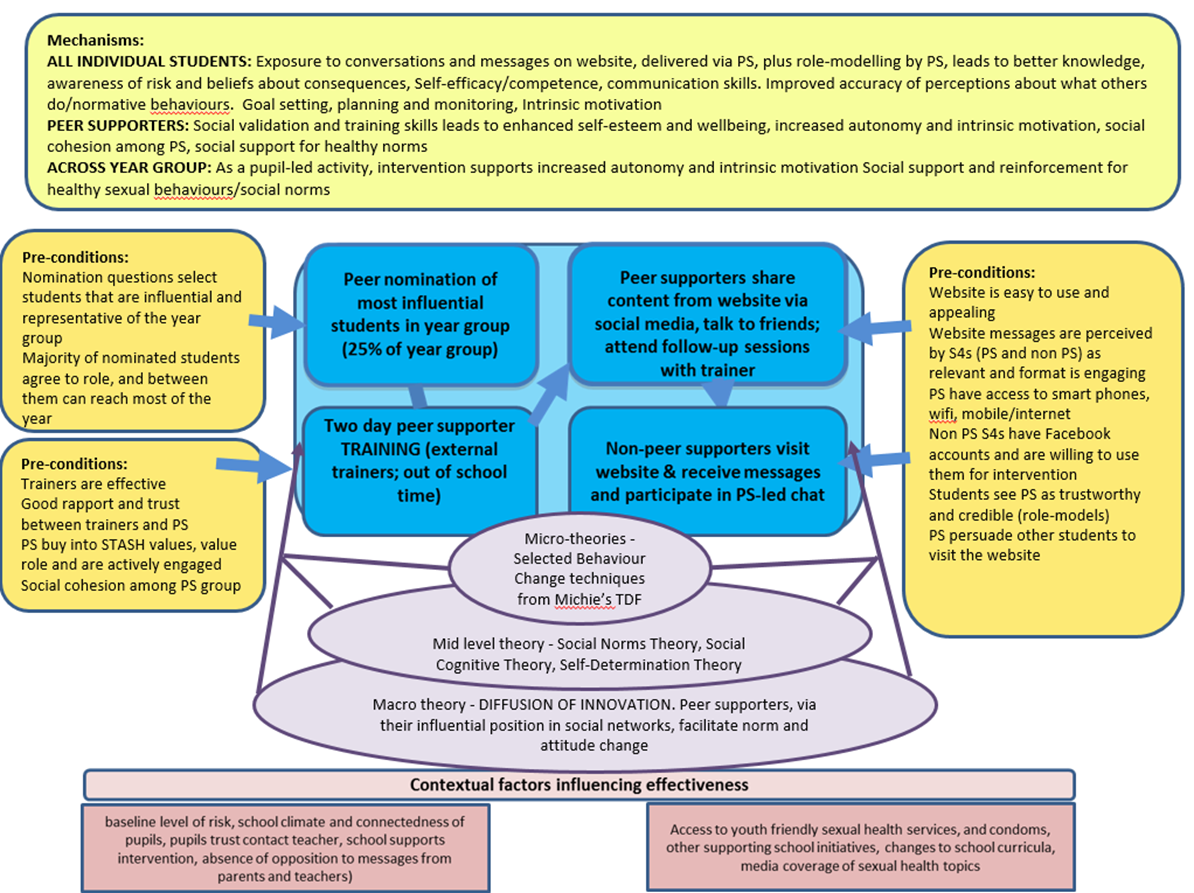
**

**Supplementary material. Programme Theory at time of Intervention Implementation.**

Reproduced from Forsyth et al.[35] This is an Open Access article distributed in accordance with the terms of the Creative Commons Attribution (CC BY 4.0) license, which permits others to distribute, remix, adapt and build upon this work, for commercial use, provided the original work is properly cited. See: http://creativecommons.org/licenses/by/4.0/. The table includes minor additions and formatting changes to the original table**.**
